# Sika Deer Velvet Antler Peptide Exerts Neuroprotective Effect in a Parkinson’s Disease Model via Regulating Oxidative Damage and Gut Microbiota

**DOI:** 10.3390/ph17070972

**Published:** 2024-07-22

**Authors:** Ying Liu, Hongyuan Li, Min Yang, Jia Guo, Zepeng Sun, Shuyue Wang, Ru Li, Xin Pang, Yumi Kim, Xiaohui Wang, Yinghua Peng

**Affiliations:** 1 Institute of Special Animal and Plant Sciences, Chinese Academy of Agricultural Sciences, Changchun 130112, China; liuying05@caas.cn (Y.L.); yangmin01@caas.cn (M.Y.); guojia@caas.cn (J.G.); szp2985312356@hotmail.com (Z.S.); 82101221263@caas.cn (R.L.); p2252345316@hotmail.com (X.P.); 2 Laboratory of Chemistry Biology, Changchun Institute of Applied Chemistry, Chinese Academy of Sciences, Changchun 130022, China; hongyuan.li@ciac.ac.cn (H.L.); xiaohui.wang@ciac.ac.cn (X.W.); 3 School of Chemistry and Life Science, Changchun University of Technology, Changchun 130012, China; abc20240620123@hotmail.com; 4 Department of Biomedical Engineering, Ulsan National Institute of Science and Technology (UNIST), Ulsan 44919, Republic of Korea; 5 School of Applied Chemistry and Engineering, University of Science and Technology of China, Hefei 230026, China

**Keywords:** Parkinson’s disease, velvet antler, peptide, oxidative stress, gut microbiota, neuroprotection

## Abstract

Parkinson’s disease (PD) is the second most common neurodegenerative disorder globally. Recognizing the potential of velvet antler in the nervous system, as shown in numerous studies, this research was aimed at evaluating the neuroprotective effects of Sika Deer velvet antler peptide (VAP), along with the underlying mechanisms in neurotoxin-induced PD models. Initially, a peptidomic analysis of the VAP, which comprised 189 varieties of peptides, was conducted using LC-MS. Nine sequences were identified as significant using Proteome Discoverer 2.5 software. In a cellular model of PD, where PC12 cells are treated with the neurotoxin 1-methyl-4-phenylpyridinium (MPP^+^), the administration of the VAP reduced the cell damage and apoptosis induced by MPP^+^. This protective effect was associated with a decrease in oxidative stress. This protective mechanism was found to be mediated through the activation of the SIRT1-dependent Akt/Nrf2/HO-1-signaling pathway. In animal models, specifically in mice with 1-methyl-4-phenyl-1,2,3,6-tetrahydropyridine (MPTP)-induced PD, the administration of the VAP effectively reduced the dopaminergic neuron damage and reversed the neurobehavioral deficits. They also diminished microglia activation and apoptosis, all without any noticeable adverse effects. Additionally, the VAP was observed to beneficially alter the gut microbiota, as marked by an increase in the abundances of *Prevotellaceae*, *Helicobacteraceae*, and *Prevotella*. These findings suggest that VAP exerts its neuroprotective effect against neurodegeneration by inhibiting oxidative stress and modulating gut microbiota.

## 1. Introduction

Parkinson’s disease (PD) is a prevalent neurodegenerative condition characterized primarily by the progressive loss of dopaminergic neurons and the accumulation of α-synuclein in the substantia nigra of the ventral midbrain [1]. The hallmark clinical symptoms of PD, including bradykinesia, static tremor, rigidity, and postural instability, stem from the depletion of dopamine-producing neurons [2]. The exact etiology of PD remains elusive, and the precise mechanisms that drive this disease are yet to be fully understood.

A critical aspect of PD pathogenesis is oxidative stress, which results from an imbalance between the generation of reactive oxygen species (ROS) and the body’s antioxidative defenses [3]. Elevated ROS levels are often linked to mitochondrial dysfunction in PD. Mitochondria maintain pathogen-associated molecular patterns and release damage-associated molecular patterns, which potentially trigger innate immune responses and further elevate oxidative stress levels [4]. Defects in mitochondrial function contribute to protein oxidation, aggregation, and apoptosis [5], and are tightly linked to genetic PD and alpha synuclein accumulation [6]. Additionally, increased oxidative stress and reduced free radical scavenging capabilities exacerbate α-synuclein aggregation in animal models [4].

Recently, interaction between the gastrointestinal environment and CNS function has been recognized as crucial for maintaining their homeostasis bidirectionally. An imbalance in gut microbiota, or dysbiosis, can lead to low-grade inflammation [7], cellular degeneration [8], and an altered state of cellular energy, culminating in increased oxidative stress [9]. Certain gut microbiota members might produce neurotoxins targeting mitochondria within the enteric and central nervous systems, leading to neurodegeneration, including PD [10]. Recent studies have reported alterations in gut microbiota and intestinal metabolism in PD patients, which affects brain function via the microbiota–gut–brain axis [11]. Specifically, decreased levels of certain microbiota, like *Prevotellaceae*, *Faecalibacterium*, and *Lachnospiraceae*, in PD patients suggest their potential protective role against the disease [12]. Similarly, changes in microbiome composition were observed in chemically induced PD models. For instance, epigallocatechin-3-gallate (EGCG) [13] and fisetin [14] have shown potential in ameliorating PD symptoms and remodeling gut microbiota in respective models.

Current treatments for PD primarily focus on symptom relief and include medications like levodopa [15], dopamine agonists [16], catechol-O-methyltransferase inhibitors [17], Monoamine oxidase type B (MAO-B) inhibitors [18], and anti-cholinergic drugs [19]. However, these treatments are still not enough to halt or slow down the neurodegenerative process of PD. Velvet antler from the sika deer (*Cervus nippon*) is used in traditional Chinese medicine and modern nutraceuticals for its benefits in treating chronic diseases and providing tonic effects. These benefits are due to its antioxidant capabilities [20], anti-inflammatory effects [20], anti-osteoporosis properties [21], and tissue repair functions [22,23]. Velvet antler methanol extracts have protective properties against oxidative stress and can alleviate symptoms in PD models [24]. Additionally, peptides derived from velvet antler can positively impact D-galactose-induced aging in mice [25] and hypoxic-ischemic injuries in rats [26], as well as show improvements in gut microbiota.

Herein, this study aimed to analyze the neuroprotective potential of velvet antler peptide (VAP) on PD models in vitro and in vivo and assess its impact on gut microbiota. In cellular and animal PD models, the VAP mitigated neurotoxin-induced damage and oxidative stress through the SIRT1-Akt/Nrf2/HO-1 pathway, improved gut microbiota, and exhibited no adverse effects, highlighting its potential as a neuroprotective agent.

## 2. Results and Discussions

### 2.1. Peptidomic Characterization of VAP

**Figure 1:**
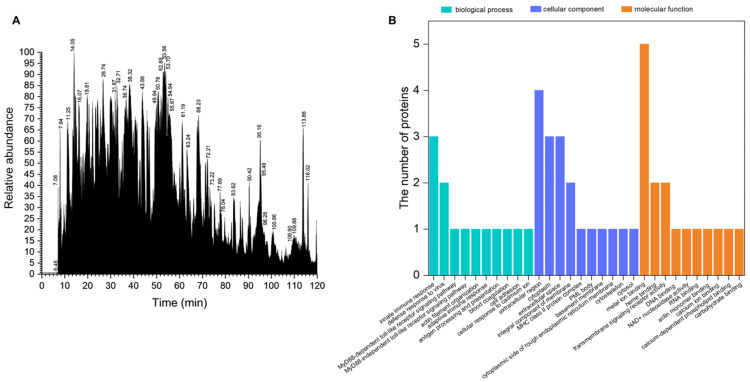
Peptidomics analysis of the VAP. (**A**) LC-MS TIC chromatographs of the VAP. (**B**) GO categorization of the sequences identified in the VAP. TIC, total ion current; GO, gene ontology.

The LC-MS total ion current (TIC) chromatograph of VAP revealed 189 varieties of peptides (Figure 1A, Table S1). The peptides from VAP were enriched in biological processes, such as the innate immune response and defense response to virus (Figure 1B). These peptides were predominantly located in the extracellular region and cytoplasm, with notable metal-binding properties (Figure 1B).

Utilizing Proteome Discoverer software, nine reliable proteins were identified in the VAP, as listed in Table 1. Notably, proteins related to molecular functions, such as metal ion binding, carbohydrate binding, and complement activation, showed higher coverage compared with other proteins. Particularly, metal ion binding proteins were abundant, with five varieties identified.

The velvet antler peptide, which is a sequence with 32 amino acid residues (VLSAT DKTNV LAAWG KVGGN APAFG AEALE RM), demonstrated a neuroprotective effect against MPP+-induced cytotoxicity in SH-SY5Y cells. When aligning this sequence with peptides from the VAP peptidome, we identified two proteins—adult beta-globin (A0A220IG97) and catalase (G0Z3A2)—with similarity percentages of 50.00% and 54.55%, respectively. Additionally, one protein identified in the VAP peptidome, 14-3-3 protein epsilon (A0A2R4PCY0), showed a notable 100.00% similarity to the CNT14 peptide (EPTVLDEVCLAHGP) from velvet antler, which is known for its role in stimulating HT22 cell growth.

### 2.2. Neuroprotective Effects of VAP in PC12 Cells Exposed to MPP^+^

**Figure 2:**
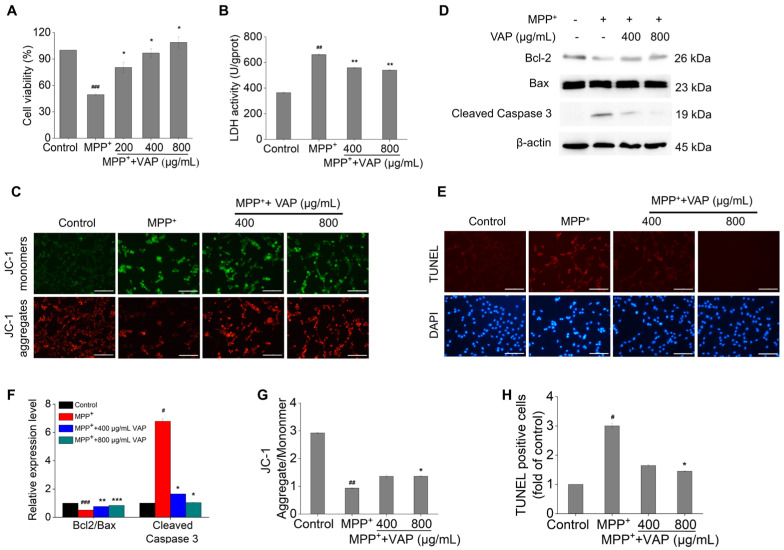
VAP protects PC12 cells from MPP^+^ induced damage and apoptosis. (**A**) Post 24 h treatment with VAP and MPP^+^, PC12 cell viability was evaluated using the WST-1 assay. (**B**) LDH release was quantified in PC12 cells treated with VAP and MPP^+^ for 24 h using an LDH assay kit. (**C**) After the 24 h treatment with VAP and MPP^+^, the mitochondrial membrane potential in PC12 cells was assessed using a JC-1 probe. Scale bar: 100 μm. (**D**) The expression levels of apoptosis-related proteins Bcl-2, Bax, and cleaved caspase-3 were determined in PC12 cells post-24 h treatment using Western blotting. β-actin served as the internal control. The relative expression of each protein was quantified based on normalization to GAPDH level and is shown on the right. (**E**) TUNEL staining (red) was conducted to detect the apoptosis in PC12 cells treated with VAP and MPP^+^ for 24 h, with DAPI staining (blue) for nuclei. Scale bar: 100 μm. (**F**) Quantification of Bcl-2/Bax and cleaved caspase-3 expression. (**G**) Quantification of Δ*ψ*m was expressed as a ratio of JC-1 aggregate to monomer fluorescence intensity. (**H**) Quantification of TUNEL-positive cells. Data are presented as mean ± SEM (n = 3). Statistical significance is indicated as ^#^ *p* < 0.05, ^##^ *p* < 0.01, and ^###^ *p* < 0.001 versus the control group; * *p* < 0.05, ** *p* < 0.01, and *** *p* < 0.001 versus the MPP^+^-treated group.

PC12 cells respond to nerve growth factor (NGF) by differentiating into cells that exhibit many characteristics of sympathetic neurons, including axonal extension. This makes them a valuable model for studying neuronal function; signaling pathways; and neurodegenerative diseases, such as Parkinson’s disease. The neurotoxin MPP^+^, which is a toxic metabolite of MPTP, is known to inhibit mitochondrial complex I activity and increase oxidative stress [27]. It is employed to induce dopaminergic neuron degeneration. PC12 cells treated with 4 mM MPP^+^ for 24 h exhibited a significant reduction in cell viability, up to 49.5%, which recapitulates previous PD model [28]. To investigate the effects of the VAP on MPP^+^-induced damage, PC12 cells were treated with 200, 400, and 800 µg/mL of VAP alongside MPP^+^. As the concentration of VAP increased, the cell viability significantly improved, where it reached 80.4%, 96.7%, and 108.7% compared with the control, respectively (Figure 2A). These results demonstrate that the VAP had a protective effect on cell viability against MPP^+^-induced cell death.

Further analysis revealed that MPP^+^ significantly increased the lactate dehydrogenase (LDH) activity, which is a marker of cell damage [29], which was mitigated by the VAP (Figure 2B). Additionally, mitochondrial membrane potential (Δ*ψ*m), which is a key indicator of mitochondrial health, was monitored using JC-1 staining [30]. The loss of Δ*ψ*m observed in MPP^+^-treated cells was significantly reduced by the VAP treatment, suggesting its role in preserving mitochondrial function (Figure 2C,G).

Apoptosis, which is a critical factor in PD progression, was assessed through the expression of apoptosis-related proteins, like Bcl-2, Bax, and caspase-3. The treatment of VAP reversed the MPP^+^-induced decrease in the Bcl-2/Bax ratio and inhibited the increase in cleaved caspase-3 expression (Figure 2D,F), highlighting its potential to counteract MPP^+^-induced apoptotic cell death.

TUNEL staining further confirmed these findings, showing a significant reduction in MPP^+^-induced apoptosis in cells treated with VAP (Figure 2E,H). Overall, these results suggest that the VAP effectively protected against MPP^+^-induced damage and apoptosis in PC12 cells, offering potential neuroprotection in PD.

### 2.3. VAP Reduces MPP^+^-Induced Oxidative Stress in PC12 Cells

**Figure 3:**
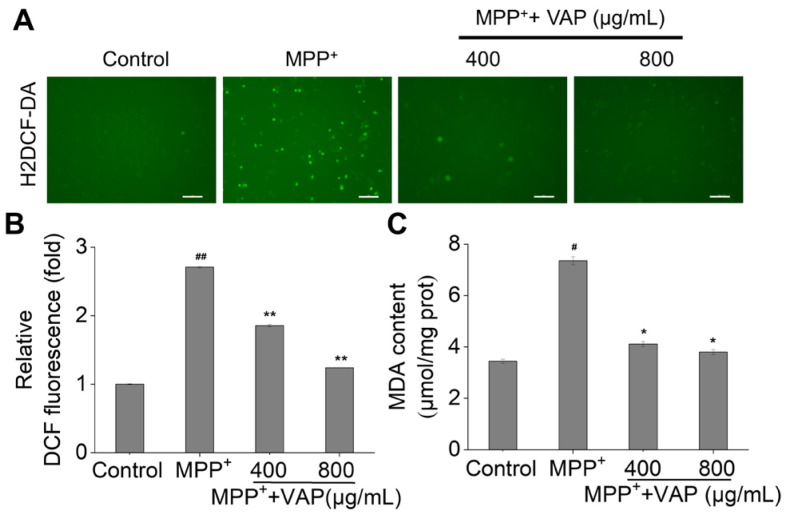
VAP reduced the MPP^+^-induced oxidative stress in the PC12 cells. The PC12 cells were treated with the VAP and MPP^+^ for 24 h. (**A**) The generation of ROS was measured using H_2_DCF-DA staining. Scale bar: 100 μm. (**B**) Quantitative analysis of the ROS production shown in (**A**). (**C**) The effect of the VAP on the MDA content was evaluated. The data are presented as the mean ± SEM (n = 3). Statistical significance is denoted as ^#^ *p* < 0.05 and ^##^ *p* < 0.01 versus the control group; * *p* < 0.05 and ** *p* < 0.01 versus the MPP^+^-treated group.

Oxidative stress, which is primarily caused by an overproduction of ROS and reactive nitrogen species, is a key pathogenetic factor in PD, leading to disrupted energy metabolism and cell apoptosis [31]. MPP^+^ induces mitochondrial dysfunction by inhibiting complex I activity in the mitochondrial electron transport chain, resulting in ROS accumulation within cells. To evaluate the impact of the VAP on the MPP^+^-induced ROS production, ROS levels in the PC12 cells were measured using DCFH-DA staining [32]. Compared with untreated control cells, there was a notable increase in green fluorescence, which indicated ROS accumulation in the cells treated with MPP^+^ (Figure 3A). However, the application of VAP significantly prevented the MPP^+^-induced increase in green fluorescence. Quantitatively, the MPP^+^ exposure led to a 2.7-fold increase in the intracellular ROS levels, which was reduced to 1.9- and 1.2-fold following the co-treatment with 400 and 800 µg/mL of VAP, respectively (Figure 3B). This suggests that VAP effectively decreased the ROS production induced by the MPP^+^ in PC12 cells.

A similar phenomenon was observed with MDA, which is another indicator of oxidative stress [33]. The MPP^+^ treatment increased MDA levels up to 7.4 µmol/mg protein, but the co-treatment with 400 and 800 µg/mL of VAP reduced these levels to 4.1 and 3.8 µmol/mg protein, respectively (Figure 3C). The lipid peroxidation assay results further supported the idea that VAP mitigated the increase in MDA levels induced by the MPP^+^, which demonstrated a dose-dependent protective effect against oxidative stress. Altogether, the MPP^+^-induced ROS could be effectively suppressed by applying the VAP in the PC12 cells.

### 2.4. Activation of the SIRT1-Mediated Akt/Nrf2/HO1 Pathway by VAP

**Figure 4:**
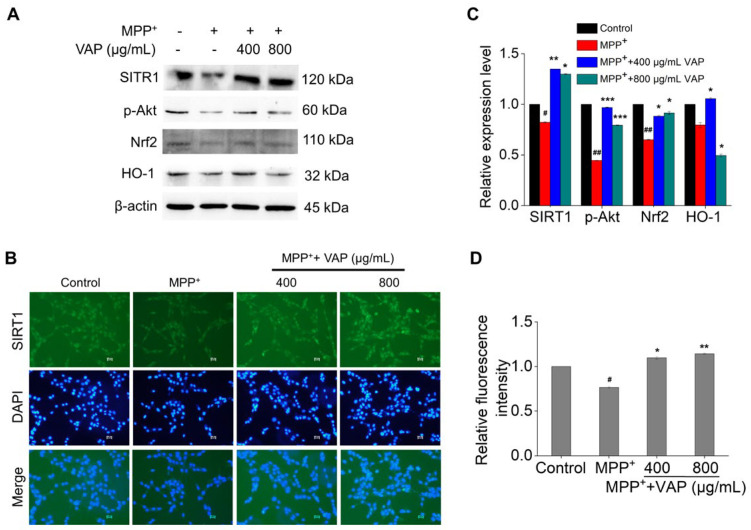
VAP protected PC12 cells against MPP^+^ by activating the SIRT1-mediated Akt/Nrf2/HO-1 pathway. (**A**) PC12 cells were treated with VAP and MPP^+^ for 24 h. SIRT1, p-Akt, Nrf2, and HO-1 were evaluated by Western blot analysis. β-actin was used as an internal control. (**B**) SIRT1 expression in PC12 cells treated with VAP and MPP^+^ for 24 h was determined by immunocytochemistry. Scale bar: 100 μm. (**C**) Quantification of SIRT1, p-Akt, Nrf2, and HO-1 expression. (**D**) Quantification of SIRT1 fluorescence intensity. The values were presented as the mean ± SEM (n = 3). Statistical significance was indicated as follows: ^#^ *p* < 0.05 and ^##^ *p* < 0.01 versus the control group; * *p* < 0.05, ** *p* < 0.01, and *** *p* < 0.001 versus the MPP^+^-treated group.

To investigate the mechanism of VAP effects on the MPP^+^-treated PC12 cells, we focused on the SIRT1-associated signaling pathways. SIRT1, which is recognized as a therapeutic target for age-related disorders, including neurodegenerative diseases, modulates ROS production and thereby reduces the oxidative stress associated with neurodegeneration [34,35]. SIRT1 activation facilitates Akt phosphorylation, mediating cell apoptosis and modulating Bcl-2 family activity, while PI3K/Akt signaling activates cellular defenses against oxidative damage through Nrf2 activation and HO-1 induction, which are both implicated in Parkinson’s disease pathogenesis [36,37]. We analyzed the protein levels of SIRT1, phosphorylated Akt (p-Akt), Nrf2, and HO-1. Twenty-four hours of MPP^+^ treatment led to the significant downregulation of these proteins, indicating increased oxidative stress and apoptosis. The treatment with VAP increased these protein levels in a concentration-dependent manner (Figure 4A,C). The immunofluorescence staining showed that the VAP reversed the expression of SIRT1 in MPP^+^-induced PC12 cells (Figure 4B,D). These results demonstrate that the VAP could upregulate the SIRT1, p-Akt, Nrf2, and HO-1 proteins in MPP^+^-induced PC12 cells, suggesting its neuroprotective role against oxidative stress and mitochondrial-mediated apoptosis through the SIRT1-mediated Akt/Nrf2/HO-1 pathway.

### 2.5. VAP Reduced PD-Related Neurological Damage in Mice

**Figure 5:**
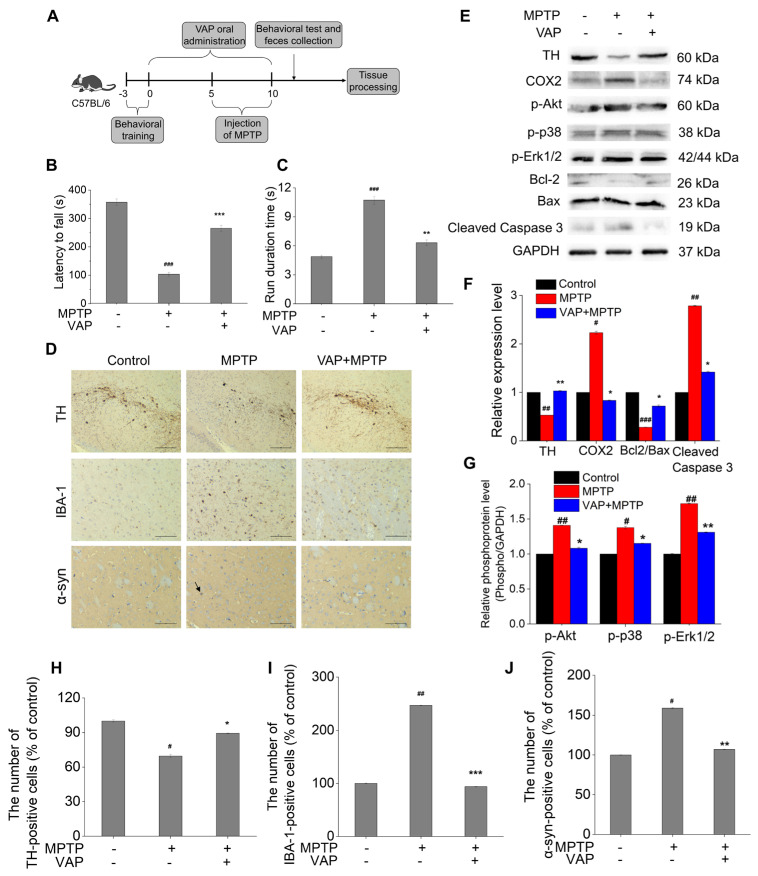
VAP reduced the PD-related neurological damage in mice. (**A**) The timeline illustrating the construction of an MPTP-induced PD mouse model and the subsequent administration of the VAP. (**B**) Rotarod test. (**C**) Climbing pole test. (**D**) Immunohistochemical staining for TH and IBA-1 in the substantia nigra and α-synuclein (α-syn) in the striatum. The black arrows indicate α-syn-positive cells. Scale bar: 100 μm. (**E**) Protein gel blot analyses of TH, COX2, p-Akt, p-p38, p-Erk1/2, Bcl-2, Bax, and cleaved caspase3 protein in the striatum, with GAPDH used as the reference. (**F**,**G**) Quantitative analysis of TH, COX2, Bcl-2/Bax cleaved caspase3, p-Akt, p-p38, and p-Erk1/2. (**H**–**J**) Number of TH-, IBA-1-, and α-syn-positive cells determined using ImageJ. The values are presented as the mean ± SEM (n = 5). Statistical significance in all analyses is denoted as follows: ^#^ *p* < 0.05, ^##^ *p* < 0.01, and ^###^ *p* < 0.001 versus the control group; * *p* < 0.05, ** *p* < 0.01, and *** *p* < 0.001 versus the MPTP-treated group.

MPTP, which is a prodrug of the neurotoxin MPP^+^, selectively targets and kills dopaminergic neurons in the substantia nigra and striatum, leading to symptoms characteristic of PD [38]. Due to its ability to deplete dopaminergic neurons and impact motor control, MPTP is frequently used in animal studies for phenocopying PD and drug discovery to treat PD. We utilized an MPTP-induced PD mouse model to investigate the in vivo effects of the VAP, as outlined in Figure 5A.

The rotarod and climbing pole tests were used to test the motor function of MPTP-treated mice. The MPTP-treated mice exhibited a reduced latency to fall (103.4 s) compared with the control group (357 s) (Figure 5B), indicating impaired motor abilities. However, this impairment was significantly mitigated in mice treated with 30 mg/kg of VAP, as evidenced by an increased latency (265.3 s). The climbing pole test results further supported these findings, showing that the MPTP-treated mice exhibited prolonged running durations (10.7 s) compared with normal mice (4.8 s), indicating a manifestation of motor dysfunction (Figure 5C). Remarkably, the treatment of VAP shortened this running duration to 6.3 s, suggesting an improvement in motor function. Collectively, these results indicate that the VAP effectively alleviated behavioral impairments in PD mice.

Tyrosine hydroxylase (TH) is essential for dopamine biosynthesis, and its expression correlates with dopaminergic neuron functionality [39]. A reduction in TH levels in brain tissues is often considered a direct sign of dopaminergic neuronal loss [39]. In the MPTP-treated group, a significant reduction in TH-positive neurons was observed in the substantia nigra, as measured by immunohistochemical staining (Figure 5D,H), indicating dopaminergic neuronal loss. The treatment with VAP significantly increased the TH-positive cells and reversed the MPTP-induced TH downregulation in the striatum, suggesting a protective effect against dopaminergic neuron loss and TH expression decrease from MPTP-induced PD. Protein gel blot analyses further supported these findings, revealing that TH expression in the striatum of PD model mice was significantly diminished compared with the control group (Figure 5E). The VAP treatment could reverse the MPTP-induced downregulation of TH expression. These results suggest that the VAP effectively counteracted the loss of dopaminergic neurons and the associated decline in TH expression induced by the MPTP in the PD model mice.

Additionally, we observed that the MPTP also induced α-synuclein accumulation in the striatum, which is a key pathology of PD, and increased the number of cells positive for IBA1 (microglial activation marker) in the substantia nigra, as measured by immunohistochemical staining (Figure 5D,I,J) [40]. The VAP treatment effectively suppressed α-synuclein accumulation in the striatum and mitigated microglial activation, as indicated by a reduced IBA-1 expression. Additionally, the COX-2 expression, which is implicated in PD neurodegeneration, was elevated in MPTP-treated mice but was inhibited in the group with VAP treatment (Figure 5E). These findings underscored the potential of the VAP in preventing neuroinflammation and dopaminergic neuron degeneration in vivo.

Mitogen-activated protein kinases (MAPKs) comprise a critical inflammatory pathway that regulates the transcription of inflammatory mediators after activation. Three subunits are present downstream of the MAPKs pathway: ERK, P38, and JNK [41]. To test whether treating with the VAP could affect the MAPKs and Akt pathway in the striatum of PD mice model, we measured the phosphorylation of proteins in the MAPKs/Akt pathway. The phosphorylation of proteins in the MAPKs/Akt pathway were significantly increased in the PD mice model, but their increases were suppressed with the VAP treatment (Figure 5E–G). The results showed that the VAP inhibited the elevated phosphorylation of Erk1/2, p38, and Akt in the PD mice.

Furthermore, biochemical analyses highlighted the function of the VAP in modulating apoptosis pathways. The MPTP treatment resulted in a decrease in Bcl2 expression, leading to a significant reduction in the Bcl2/Bax ratio and an increase in cleaved caspase-3 levels, signaling apoptosis activation. However, the VAP significantly attenuated these changes (Figure 5E). These results recapitulated the PC12 cell results (Figure 2D) and demonstrated that the VAP successfully enhanced the Bcl2/Bax ratio while inhibiting the caspase-3 activation in the striatum of the MPTP-treated mice. Consequently, the VAP appeared to play a crucial role in preventing the loss of dopaminergic neurons by mitigating apoptosis in this PD model.

**Figure 6:**
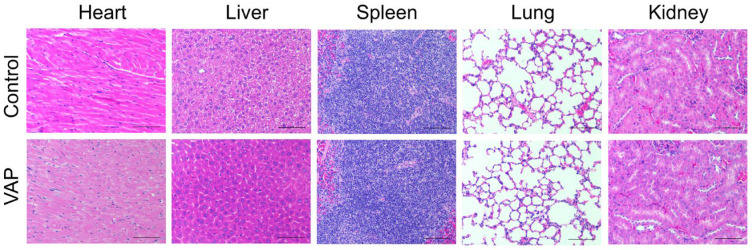
Hematoxylin and Eosin (H&E) staining of the different organs (heart, liver, spleen, lung, and kidney) in mice treated with the VAP (30 mg/kg, n = 5). Scale bar: 100 μm.

**Figure 7:**
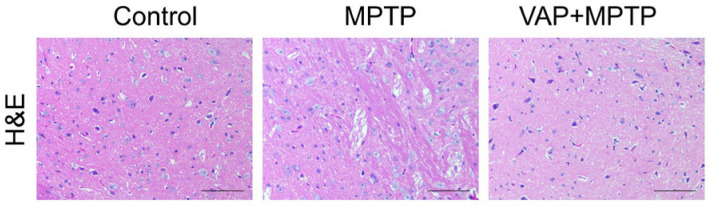
Hematoxylin and Eosin (H&E) staining of the substantia nigra in three different treated groups of mice: the saline control, MPTP-treated group, and VAP (30 mg/kg) + MPTP-treated group. Scale bar: 100 μm.

To evaluate the in vivo toxicity of the VAP, we performed histopathological analysis on mouse tissues, including the heart, liver, kidney, spleen, lung, and substantia nigra. The tissue sections from the mice treated with VAP displayed no signs of tissue damage in all the organs we tested (Figure 6). In contrast, the MPTP-treated mice exhibited noticeable atrophy, where the neurons and glial cells in the substantia nigra appeared distinctly separated (Figure 7). However, this tissue separation and atrophy were not observed in the substantia nigra of the mice treated with VAP, indicating tissue normalization. These findings suggest that the VAP was not only effective in treating PD but also safe, as it did not cause any apparent tissue damage in the tested mice.

### 2.6. VAP Improved MPTP-Induced Gut Microbiota Dysbiosis

Recent research suggests a connection between gut microbiota and the development of PD, with changes in the gut microbial composition observed in PD patients and animal models. Notably, the family *Prevotellaceae*, which is linked to PD severity and progression [42], and the genus *Prevotella*, which is important for maintaining gut mucosal integrity, were found to decrease in PD. These alterations in gut microbiota are considered potential biomarkers and therapeutic targets for PD.

**Figure 8:**
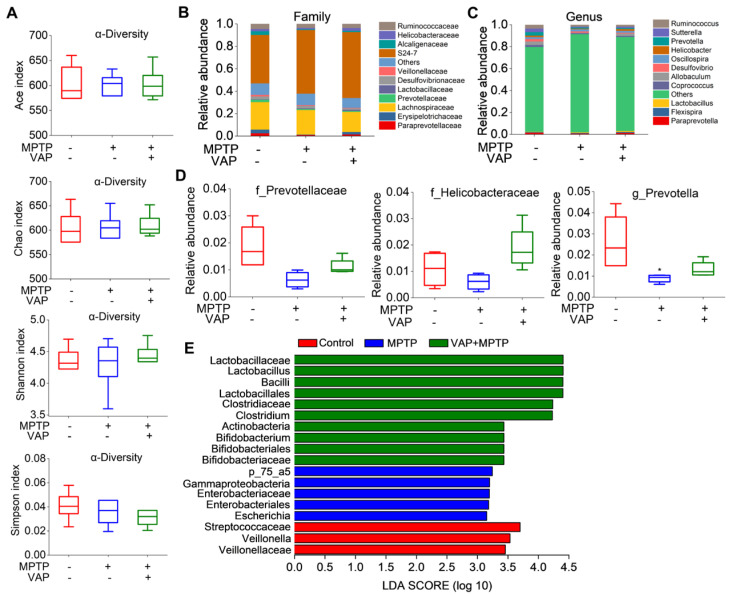
VAP improved the MPTP-induced gut microbiota dysbiosis in the mice. (**A**) Alpha diversity analysis of the gut microbiota showed no significant differences between the mice from the three different groups. (**B**,**C**) Barplots of the relative abundance of the three different groups at the family level (**B**) and genus level (**C**). (**D**) The relative abundances of f_*Prevotellaceae*, f_*Helicobacteraceae*, and g_*Prevotella* among the three different groups. * *p* < 0.05 compared with the control group. (**E**) LEfSe analysis identified specific bacterial taxa that acted as biomarkers in each group. The cladogram highlighted the control-group-enriched taxa (red), MPTP-induced PD-group-enriched taxa (blue), and VAP-treated PD-group-enriched taxa (green). Only taxa with an LDA score greater than 2 are displayed, emphasizing the most significant differences in microbial composition between the groups.

In our study, we utilized 16S rRNA sequencing and bioinformatic analysis to assess the impact of VAP on the gut microbiota of MPTP-induced PD mice. The alpha diversity indices, including the Ace, Chao, Shannon, and Simpson indices, showed no significant differences between the groups (Figure 8A). However, taxonomic classification analysis of the OTUs revealed considerable changes in microbiota composition in MPTP-treated mice and MPTP-VAP-treated mice (Figure 8B,C). Notably, the relative abundances of the *Prevotellaceae* and *Helicobacteraceae* families were lower in the MPTP-treated group than in the control group. In contrast, the treatment with application of VAP led to an increase in these taxa compared with the MPTP group (Figure 8D). Similarly, at the genus level, the *Prevotella* abundance was significantly reduced in the MPTP group but increased following the VAP treatment.

The gut microbiota composition was further analyzed using the LDA effect size (LEfSe) method. The cladogram (Figure 8E) illustrates the structure of the gut microbiota and highlights the predominant bacteria in each group, with significant differences between the taxa emphasized. Our findings indicate that the PD mice exhibited gut microbial dysbiosis, and the VAP appeared to modulate the microbiota composition, potentially offering beneficial effects for PD mice induced by the MPTP.

Some compounds, despite their large molecular size, have shown potential in treating brain diseases through the gut–brain axis. The gut–brain axis is a complex communication network linking the gastrointestinal tract and the central nervous system, mediated by neural, hormonal, and immunological pathways. Here, the VAP influenced this axis by modulating the gut microbiota composition, where it may enhance the production of beneficial metabolites. These changes in the gut environment can help to maintain the integrity of the gut barrier, preventing the translocation of pro-inflammatory cytokines and other harmful substances that could trigger neuroinflammation and contribute to the progression of brain diseases. Thus, targeting the gut–brain axis offers a promising therapeutic strategy in the treatment of neurological disorders.

## 3. Materials and Methods

### 3.1. Materials

Velvet antler from sika deer (*Cervus nippon*) was provided by the Zuojia Sika Deer Farm (Jilin, China). Preparation of velvet antler peptide: velvet antler powder was mixed with distilled water at the proportion of 1:10 and shaken thoroughly for 24 h in an ice bath. The supernatant was obtained after centrifugation at 8000× *g* for 15 min and then frozen dry by a lyophilizer (BILON FD-2, Shanghai, China). The yield of VAP was 12% (*w*/*w*) of the dried sample. 1-methyl-4-phenylpyridine (MPP^+^, D048) and 1-methyl-4-phenyl-1,2,3,6-tetrahydropyridine (MPTP, M0896) were obtained from Sigma-Aldrich (St. Louis, MO, USA). Bcl2 (#4223), cleaved caspase3 (#9664), β-actin (#8457), GAPDH (#5174), COX2 (#412282), and α-syn (#4179) antibodies were obtained from Cell Signaling Technology (Beverly, MA, USA). Bax (ab32503), SIRT1 (ab189494), Nrf2 (ab137550), HO-1 (ab13248), TH (ab112), p-p38 (ab4822), and p-Erk1/2 (ab76299) antibodies were obtained from Abcam (Cambridge, MA, US). p-Akt (ARG51558) and IBA-1 (ARG63338) were purchased from Arigo biolaboratories (Shanghai, China). The horseradish peroxidase (HRP)-conjugated secondary antibodies (NA934 and NA931) were purchased from Cytiva (Shanghai, China).

### 3.2. Peptidomic Analysis

A total of 150 μg of VAP was taken and placed in a water bath at 37 °C for enzymatic hydrolysis overnight (18–20 h). After desalting, the supernatant was taken for nanoliter liquid chromatography (EASY-nLC 1200, Thermo Fisher Scientific, Waltham, MA, USA) separation and Q-Exactive high-resolution mass spectrometer (Q-Exactive HF, Thermo Fisher Scientific, Waltham, MA, USA) detection.

For the liquid chromatography, two types of separation solutions were used: liquid A used in the liquid phase was a 0.1% formic acid aqueous solution, and liquid B was a 0.1% formic acid acetonitrile aqueous solution (acetonitrile is 80%). The liquid chromatography column (50 μm × 150 mm, Acclaim PepMapTM, Thermo Scientific Technology Inc., Waltham, MA, USA) was equilibrated with 92% A solution, the injection volume of 1 μL was separated by the column, and the relevant liquid gradient settings were as follows: 0 min–98 min, the linear gradient of liquid B went from 8% to 28%; 98–113 min, the linear gradient of liquid B went from 28% to 37%; 113–117 min, the linear gradient of liquid B went from 37% to 100%; 117–120 min, solution B maintained at 100%.

In the mass spectrometry analysis, the following parameters were set: For the primary scan, the resolution was set at 60,000, with an AGC target of 3 × 10^6^, a maximum IT of 40 ms, and a scan range of 400 to 1800 *m*/*z*. For the secondary scan, the resolution was 15,000, AGC target was 1 × 10^5^, maximum IT was 60 ms, TopN was set to 20, and NCE/stepped NCE was 27.

### 3.3. LC-MS Data Processing and Gene Ontology (GO) Analysis

The LC-MS data processing was conducted using Proteome Discoverer 2.5 software with specified parameters: fixed modification was carbamidomethyl (C) and variable modification was oxidation (M). Trypsin was set as the enzyme, allowing for a maximum of two missed cleavages. The peptide mass tolerance was configured at 10 ppm, and the fragment mass tolerance was configured at 0.02 Da using monoisotopic mass values. The significance threshold was set at 0.05. Subsequently, GO analysis of the identified VAP was performed utilizing RStudio.

### 3.4. Cell Viability Assay

PC12 cell line was purchased from Wuhan Pricella Biotechnology Co., Ltd. (CL-0481, Wuhan, China). PC12 cells were cultured in Dulbecco’s modified Eagle’s medium (DMEM) containing 2.5% FBS, 15% HS, 4 mM glutamine, 100 U/mL penicillin, and 100 μg/mL streptomycin at 37 °C in a 5% CO_2_ incubator. PC12 cells were exposed to different treatments, as indicated. After 24 h of treatment, 20 μL of WST-1 solution (KeyGEN Bio TECH, Nanjing, China) was added to each well and the plates were further incubated for 2 h at 37 °C. Absorbance at 450 nm was measured using a microplate reader (Bio-Tek synergy H1, Winooski, VT, USA).

### 3.5. LDH Assay

PC12 cells were exposed to different concentrations for 24 h, as indicated. The activity of LDH in the cell lysate was determined using a commercial kit (Nanjing Jiancheng Bioengineering Institute, Nanjing, China) according to the manufacturer’s protocol.

### 3.6. Measurement of Mitochondrial Membrane Potential

Mitochondrial membrane potential (Δ*ψ*m) of PC12 cells was determined using a JC-1 fluorescent dye (Beyotime Biotechnology, Shanghai, China). Cells were stained with JC1 probe in the dark at 37 °C for 20 min, washed twice with PBS, and observed under a Nikon TS2-FL fluorescent microscope (Tokyo, Japan). The level of Δ*ψ*m was calculated as the JC-1 aggregate/monomer fluorescence intensity ratio.

### 3.7. TUNEL Staining

Detection of DNA fragments was carried out with the one-step TUNEL apoptosis assay kit (Beyotime Biotechnology, Shanghai, China). Briefly, cells were fixed with 4% paraformaldehyde in PBS, permeabilized with 0.3% Triton X-100, and washed with PBS. Cells were then incubated with TUNEL reaction mixture for 1 h at 37 °C in the dark. Then, the nucleus was stained with 10 μg/mL of DAPI for 5 min. Finally, the cells were washed with PBS 3 times before imaging. Quantification was carried out using ImageJ v1.51 software.

### 3.8. Measurement of ROS

Intracellular ROS levels were analyzed using a ROS assay kit (Beyotime Biotechnology, Shanghai, China). PC12 cells were washed three times, and then incubated with 10 µmol/L DCFH-DA at 37 °C for 20 min. Images were obtained using a fluorescent microscope. Quantification of the fluorescence intensity was measured with a microplate reader (Bio-Tek synergy H1, Winooski, VT, USA).

### 3.9. Measurement of Lipid Peroxidation

PC12 cells were treated with VAP (400 or 800 μg/mL) and 4 mM MPP^+^ for 24 h. After treatments, cells were harvested by centrifugation for 5 min at 1000 rpm. The levels of malondialdehyde (MDA) were evaluated using a commercial kit (Beyotime Biotechnology, Shanghai, China) according to the manufacturer’s instructions.

### 3.10. Western Blotting

The samples were first resolved in 12% sodium dodecyl sulfate-polyacrylamide gel electrophoresis (SDS-PAGE) and then transferred to nitrocellulose membranes, followed by blocking membranes with 5% nonfat dry milk in TBST buffer (25 mM Tris-HCl, 140 mM NaCl, 0.05% Tween 20, pH 7.5) for 1 h and incubation with the appropriate primary antibody (0.5 μg/mL) at 4 °C overnight. Blots were washed five times using TBST solution, and the membrane was incubated with HRP-conjugated secondary antibody (50 ng/mL) for 1 h at room temperature. After sufficient washing, antibody complexes attached to the membrane were visualized by a Tanon-4600 SF (Tanon, Shanghai, China) when reacting with SuperSignal West Pico PLUS Chemiluminescent Substrate (34580, Thermo Scientific, Waltham, MA, USA). ImageJ was used for densitometric analysis.

### 3.11. Immunocytochemistry

PC12 cells were exposed to different treatments, as indicated. After 24 h treatment, cells were fixed with 4% paraformaldehyde. Then, 1% bovine serum albumin (BSA) in PBS was used for blocking for 30 min. The cells were incubated overnight at 4 °C with SIRT1 primary antibody diluted 1: 250 in 1% BSA in PBS. After five washes with PBS, the cells were incubated with a secondary Alexa Fluor 488-conjugated anti-rabbit IgG (HS131, TransGen, Bejing, China) for 1 h at room temperature. The cellular nucleus was stained with DAPI (C0065, Solarbio, Bejing, China) for 5 min. The staining of PC12 cells was visualized by a Nikon TS2-FLfluorescence microscope (Tokyo, Japan). Quantification was carried out using ImageJ v1.51 software.

### 3.12. Animals and Treatment

Male C57BL/6 mice were purchased from Liaoning Changsheng Biotechnology (Benxi, China). The mice (8–10 weeks old, weighing 20–25 g) were used for this study. The mice were kept in a temperature-controlled room (22 ± 1 °C) at 50–60% relative humidity with a 12/12 h light–dark cycle and had free access to food and water. All the animal-handling procedures were performed in strict accordance with the regulations for the Administration of Affairs Concerning Experimental Animals approved by the State Council of the People’s Republic of China (11-14-1988). The mice were randomly divided into three groups (n = 6 per group): the control group treated with the same amount PBS; the MPTP group, which received MPTP (30 mg/kg) by intraperitoneal administration; and the MPTP+VAP group, which received MPTP (30 mg/kg, i.p.) by intraperitoneal administration and 30 mg/kg of VAP by gavage administration. VAP was given daily from day 0 to day 10. The MPTP was administered daily from day 5 to day 10.

### 3.13. Rotarod Test

The rotarod test was conducted according to the previous study [43]. Briefly, the rotarod test was performed on day 10 by using the rotarod equipment (IITC Life Science, Woodland Hills, CA, USA). Before starting the experiment, a rotarod train was performed for three consecutive days at a fixed speed (40 r/min). Latency until fall was automatically recorded by magnetic trip plates. For each animal, the experiment was repeated three times, and the average time was calculated.

### 3.14. Pole Test

According to the previous study [44], a rough-surfaced plastic rod (1.5 mm in diameter, 50 cm in height) was used to make the pole. The mouse was placed on the top of the pole facing head up, and the time taken to reach the bottom of the pole was recorded. The mice received three trials at 1 min intervals, and the average times were analyzed.

### 3.15. Immunohistochemistry

The mice were euthanized by intraperitoneal injection of 50 mg/kg pentobarbital sodium. Every effort was made to minimize the suffering of the animals. The brain samples were collected. Subsequently, a 4% paraformaldehyde solution was used to fix the brain tissue. These were embedded in paraffin after dehydration and cut into 5 μm coronal sections. The sections were used for immunochemistry staining. Slices were incubated with the TH (1:200), IBA-1 (1:200), and α-syn primary antibody (1:200) overnight at 4 °C. The sections were then incubated with secondary antibody (1:400) and visualized using diaminobenzidine (DAB) (DA1010, Solarbio, Bejing, China) as the chromogen. The substantia nigra and striatum regions were defined according to the brain anatomy map. Images were captured using a light microscope equipped with a digital camera. Cells with brown granules were considered immunoreaction positive. ImageJ v1.51 software was used to analyze the images according to the manual. Images were converted to grayscale, and a threshold was applied to identify positive cells. The number of positive cells was counted. Quantification was performed blinded to the control groups to reduce the bias. Each image was analyzed three times.

### 3.16. Hematoxylin and Eosin (H&E) Staining

Tissues, including brains, hearts, livers, spleens, lungs and kidneys, were collected and fixed with 4% paraformaldehyde for 24 h at 4 °C and imbedded in paraffin. Tissue sections (3 μm) were deparaffinized and stained with H&E (G1120, Solarbio, Bejing, China). At least five paraffin sections from each tissue were used for H&E staining.

### 3.17. Gut Microbiota Analysis

The fresh fecal samples of all mice were collected and stored at −80 °C. The fecal bacterial composition was determined by amplifying and sequencing the V3 and V4 regions of the bacterial 16S rRNA. Microbial DNA was extracted from the fecal samples (Bioer Technology, Hangzhou, China). 16S rRNA gene amplification was evaluated using the Illumina MiSeq platform (BGI, Shenzhen, China). In this study, operational taxonomic units (OTUs) served as the basis for calculating the alpha diversity indices, including Ace, Chao, Shannon, and Simpson. To assess the characterization of the differences between these OTUs, linear discriminant analysis (LDA) effect size (LEfSe) was employed. LEfSe utilizes the Kruskal–Wallis rank-sum test to identify features (OTUs) that exhibit significantly different abundances between groups. Subsequently, LDA is applied to estimate the effect size of each identified feature, providing a measure of its relative impact.

### 3.18. Statistical Analysis

Data were analyzed with Origin 2019b (OriginLab Corp., Northampton, MA, USA). The data conformed to a normal distribution. All values are presented as the mean ± SEM. For the analysis of experimental data, a t-test was used to compare any two groups, and one-way ANOVA was used for comparing more than two groups.

## 4. Conclusions

**Figure 9:**
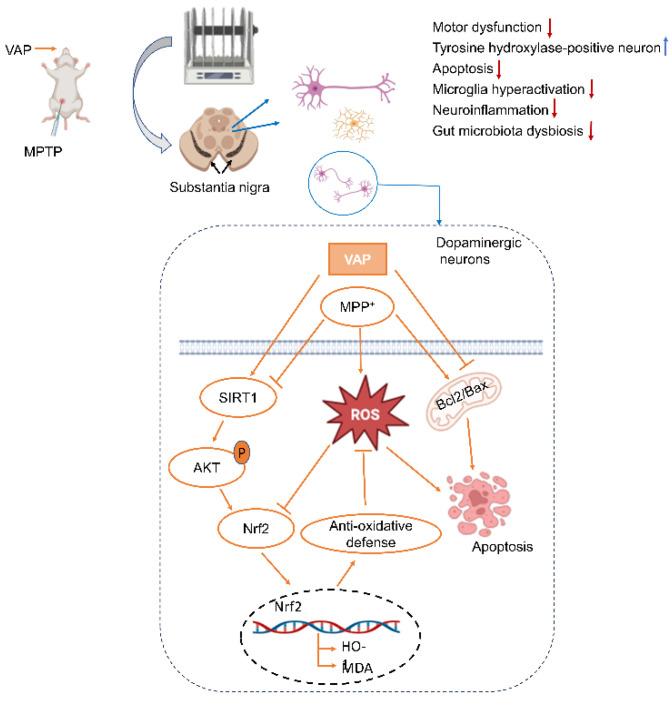
The possible pathway of PD protection induced by the VAP.

In this study, we explored the neuroprotective potential of VAP in Parkinson’s disease (PD) models. Our in vitro investigations using PC12 cells demonstrated that the VAP significantly reduced apoptosis and oxidative stress by activating the SIRT1-mediated Akt/Nrf2/HO-1 pathway, which is crucial in combating neurodegenerative processes. In the in vivo PD model, the VAP effectively alleviated dopaminergic neuron loss in mice. Additionally, the VAP played a significant role in modulating gut microbiota dysbiosis, which is a factor that is increasingly recognized in PD pathogenesis (Figure 9). These results collectively highlight the multifaceted neuroprotective actions of VAP. Given these promising findings, VAP emerges as a potential therapeutic agent for PD, offering a novel approach to address both neuronal health and gut microbiota balance. Our study paves the way for further research into its clinical applications and underlying mechanisms in PD management.

## Figures and Tables

**Table 1:** Proteins of VAP by LC-MS identification.

Protein IDs	Peptides	Unique Peptides	Sequence Coverage [%]	Mol. Weight [kDa]	Sequence Length	GO Molecular Function
X2GM95	74	31	85	66.1	583	Metal ion binding
A0A2S1M4Y6	51	8	67	68.8	607	Metal ion binding
A0A220IG97	22	22	87	15.9	145	Metal ion binding
A0A385XR53	10	10	86	14.7	135	Carbohydrate binding
Q9N0M4	10	10	53	18.7	167	Complement activation
V5LTF3	6	6	50	15.6	151	Metal ion binding
G0Z3A2	14	14	39	60	527	Metal ion binding
A0A2R4PCY0	13	13	43	29.1	255	Phosphoserine residue binding
J9UJQ1	11	11	40	38.6	339	Calcium ion binding

## Data Availability

Data are contained within the article and Supplementary Materials.

## Supplementary Materials

The following supporting information can be downloaded at: https://www.mdpi.com/article/10.3390/ph17070972/s1, Table S1. Peptides of VAP by LC-MS identification.
